# Use of the socio-ecological model to explore factors that influence the implementation of a diabetes structured education programme (EXTEND project) inLilongwe, Malawi and Maputo, Mozambique: a qualitative study

**DOI:** 10.1186/s12889-021-11338-y

**Published:** 2021-07-08

**Authors:** C. Bamuya, J. C. Correia, E. M. Brady, D. Beran, D. Harrington, A. Damasceno, A. M. Crampin, Ana Magaia, Naomi Levitt, M. J. Davies, M. Hadjiconstantinou

**Affiliations:** 1 Malawi Epidemiology and Intervention Research Unit, Lilongwe, Malawi; 2 Unit of Patient Education, Division of Endocrinology, Diabetology, Nutrition and Patient Education, WHO Collaborating Center, Department of Medicine, University of Geneva and Geneva University Hospitals, Geneva, Switzerland; 3 University Hospitals of Leicester NHS Trust, Leicester Diabetes Centre, Leicester, UK; 4 Department of Cardiovascular Sciences, University of Leicester, Leicester, UK; 5 Division of Tropical and Humanitarian Medicine, Faculty of Medicine, University of Geneva and Geneva University Hospitals, Geneva, Switzerland; 6 Diabetes Research Centre, College of Life Sciences, University of Leicester, Leicester, UK; 7 Psychological Sciences and Health, University of Strathclyde, Glasgow, Scotland; 8 Faculty of Medicine, Eduardo Mondlane University, Maputo, Mozambique; 9 The University of Cape Town, Cape Town, South Africa

## Abstract

**Background:**

Diabetes Self-Management Education and Support (DSMES) programmes are vital for type 2 diabetes mellitus (T2DM) management. However, they are limited in Sub-Saharan Africa (SSA). To address this gap, a DSMES, namedEXTEND was developed in Lilongwe (Malawi) and Maputo (Mozambique). This qualitative study aimed to explore factors that influence the implementation of DSMES in these settings.

**Methods:**

The Socio-ecological model was applied to explore factors influencing the implementation of DSMES in SSA. Data was analysed using the Framework method and constant comparative techniques. Sixty-six people participated in the study: people with T2DM who participated in the EXTEND programme; healthcare professionals (HCPs), EXTEND educators, EXTEND trainers, and stakeholders.

**Results:**

Our findings indicate that there is a need to develop an integrated and dedicated diabetes services in SSA healthcare systems, incorporating culturally adapted DSMES and tailored diabetes training to all professions involved in diabetes management. Traditional media and the involvement of community leaders were proposed as important elements to help engage and promote DSMES programmes in local communities. During the design and implementation of DSMES, it is important to consider individual and societal barriers to self-care.

**Conclusion:**

Findings from this study suggest that multi-faceted factors play a significant role to the implementation of DSMES programmes in LICs. In the future, EXTEND could be incorporated in the development of diabetes training and dedicated diabetes services in SSA healthcare systems, acting as an educational tool for both people with T2DM and HCPs. This project was supported by the Medical Research Council GCRF NCDs Foundation Awards 2016 Development Pathway Funding.

**Supplementary Information:**

The online version contains supplementary material available at 10.1186/s12889-021-11338-y.

**Table:** 

**Summary Box** **What is already known?** • The prevalence of T2DM in Malawi and Mozambique is rising. • Factors that affect the successful management of T2DM in SSA include poor health systems and a lack of resources including infrastructure and negative traditional attitudes towards T2DM management. **What are the new findings?** • We found that there is a large unmet need for dedicated DSMES guidelines and diabetes policies in SSA countries. • The involvement of community settings and community leaders could help build a strong infrastructure for the delivery of culturally adapted and community-led DSME programmes. • Cultural beliefs and attitudes are major determinants that may influence the understanding and management of T2DM. • During the development and implementation of DSMES, it is important to consider people’s personal necessities and available resources; and to address misconceptions derived from societal beliefs and stigma that accompany diabetes management. **What do the new findings imply?** • There is a need to develop an integrated and dedicated diabetes services in SSA healthcare systems, incorporating culturally adapted DSMES and tailored diabetes training to all professions involved in diabetes management. • We must ensure that a ‘shared language’ is adopted across SSA and endorsed by national guidelines specifically for DSMES.	

## Background

Globally, it is estimated that 463 million adults live with diabetes [1], of which 90% are type 2 diabetes mellitus (T2DM) [2]. T2DM is fast becoming a prominent cause of high levels of morbidity and mortality in low-income countries (LICs) in sub-Saharan African (SSA) [3]. The prevalence of T2DM in Malawi and Mozambique is 2.6% [4] and 3.3% [5] respectively. Although the prevalence of T2DM in the aforementioned countries are below the global average of T2DM, these rates, are nonetheless, equally alarming and must be addressed with caution. T2DM management requires a healthy lifestyle and optimal medication regimen [2]. Factors that affect the successful management of T2DM in SSA include poor health systems and a lack of resources including infrastructure and negative traditional attitudes towards T2DM management [6, 7]. Issues around poor patient outcomes for diabetes are multi-facetted and are largely associated with five distinct domains that include individual (factors and or circumstances directly related to the person with diabetes such as their occupation, their health literacy, their existing co-morbidities etc.); interpersonal (the person with diabetes relationship(s) with spouse, carer, consultant, children etc.); community (the impact of the local community on the person with diabetes, i.e. access to green space, community groups/activities); policymakers (those who decide what provisions should be prioritised for diabetes care or the person with diabetes) and commissioners (those who decide what provisions should be paid for/provided to diabetes care or the person with diabetes) [8, 9].

This implies that decisions impacting successful diabetes self-management education and support (DSMES) programmes, are influenced greatly by the infrastructure available in the communities [10]. Therefore, in such settings potential cost-effective and effective strategies that improve self-management are of paramount importance. DSMES is recognised as an essential component of diabetes care [3]. International recommendations for DSMES were developed to increase their effectiveness and improve patient care [3]. However, there are no DSMES that meet these recommendations in LICs in SSA [11, 12].

To address this issue, the Extending Availability of Self-management Diabetes program (EXTEND) study was developed. This study tested a culturally adapted DSMES to support people with T2DM in two SSA countries (Mozambique and Malawi) and showed effectiveness in biomedical and psychological outcomes [13]. Educators trained by UK national trainers at site, delivered EXTEND, in line with international standards for DSMES. The successful implementation of such a DSMES programme requires a multi-level approach.

We aimed to explore multi-faceted factors that influence the implementation of this DSMES programme (EXTEND) delivered in Mozambique and Malawi, to understand individual and environmental barriers necessary to establish DSMES in SSA.

## Methods

### Study design and setting

This qualitative study was part of the EXTEND study and took place in two cities: Lilongwe in Malawi and Maputo in Mozambique.

### Recruitment

The inclusion criteria were: (i) adults > 18 years and either (i) attended or delivered the EXTEND programme; or (iii) were involved in the diabetes care system of Lilongwe or Maputo or (iv) had trained as educators for the programme. Focus groups and telephone interviews were conducted with six different groups, using a snowballing recruitment technique where required:

- EXTEND participants
- EXTEND educators
- Community participants (people with T2DM who received EXTEND in their community) (only in Lilongwe)
- Local healthcare professionals (HCPs) (i.e. doctors)
- Local stakeholders and policy makers (i.e. Ministry of Health)
- Trainers who trained the educators

### Data collection

Telephone interviews (approximately 40 min) were conducted with trainers. Focus groups were conducted in the Faculty of Medicine premises (Maputo), and in Area 25 health centre (Lilongwe) (August 2018 to April 2019) with the remaining aforementioned groups, and each focus group discussion lasted approximately 90 min. The discussions allowed for detailed exploration of individual views and thoughts on barriers and facilitators to implementation of the programme.

The focus groups and interviews were carried out by our research team (MH, CB, JCC). MH, who has extensive experience in qualitative research, led data collection and analysis. Where required, research members also acted as translators (JCC). Maputo focus groups were supported by note takers (EB, DB, DH) in case of any issues with audio recordings.

Data collection followed an iterative process. The same topic guide was used for both locations, and after the first two focus groups, topic guides were refined to include a discussion of emerging themes, specifically around diabetes guidelines and HCPs training (see Supplementary File). Data was audio recorded and transcribed verbatim. In Maputo, these were translated into English from a local independent transcriber; in Lilongwe transcripts were translated by the local research team.

### Data analysis

Data were analysed using the Framework method [14], applying constant comparative techniques [15]. Taking an inductive thematic approach, transcripts were analysed by two researchers (MH and CB), who reviewed the data across both locations. An initial coding framework was generated, and further refined through additional coding against transcripts. Data were subsequently summarised and exported into matrices to enable comparison of themes systematically. Data were managed using NVivo 10 qualitative data indexing software [16].

To ensure credibility [17], we used investigator triangulation [17], whereby the two researchers (MH and CB) coded and analysed the data for both localities. Regular meetings were held during the data analysis process and discrepancies were resolved through discussion. Data saturation was achieved and sufficient information related to our research question was collected.

### Theoretical framework

The analysis of the data was underpinned by the Ssocio-ecological model, a theory based framework that explores the multifaceted and interactive effects of personal and environmental factors that determine change [18]. The rationale for this theory application was to provide us with a robust platform to enable further exploration of factors that influence adoption of DSMES in Malawi and Mozambique on an individual, community, organisation and policy level [18].

### Ethical approval

Ethical approval was obtained from the following Research Ethics Committees: the Scientific Commission of the Faculty of Medicine and the Mozambique National Research Ethics Committee; the College of Medicine, Malawi; and the University of Leicester, College of Life Sciences.

## Results

### Participants

Sixty-six individuals took part across the two sites. Two telephone interviews were carried out with the two trainers who delivered training to EXTEND educators. Six focus groups were conducted in Lilongwe (*n* = 45), and four in Maputo (*n* = 21). In Maputo this included: patients (*n* = 7 (3 (43%) males)); educators (*n* = 4); stakeholders (*n* = 2); HCPs (*n* = 6). Stakeholders consisted of representatives from the non-communicable disease (NCD) Department of the Mozambican Ministry of Health and the Eduardo Mondlane University of Mozambique; HCPs consisted of medical doctors, one internal medicine physician and one endocrinologist. Similarly, in Lilongwe the following took part: patients (*n* = 22 (11 (50%) males)); educators (*n* = 6); stakeholders (*n* = 3); HCPs (*n* = 3); community participants (*n* = 11). Stakeholders consisted of representatives from theNCD Department of the Malawi Ministry of Health; HCPs consisted of clinicians, including the deputy clinic in charge.

### Themes

Codes were categorised into five themes based on the socio-ecological framework: (i) individual influences; (ii) interpersonal influences; (iii) organisational influences; (iv) community influences; (v) public policy influences. Each theme is broken down into sub-themes for further exploration (see Table 1).

**Table 1:** Breakdown of themes identified in the EXTEND qualitative study

THEMES	SUB-THEMES
**Individual influences**	Beliefs and values
**Interpersonal influences**	Benefits to and influences of family
**Organisational influences**	Diabetes specialist training Role of deciders and the government
**Community influences**	Reach out Setting for diabetes education and EXTEND
**Policy influences**	Local guidelines on diabetes NCD funding and resources Integrated and dedicated diabetes system in the SSA healthcare system

#### Individual influences

##### Beliefs and values

Cultural beliefs appeared to influence the understanding of the disease for people with T2DM. This included the strong belief, particularly in rural areas, that the symptoms of the disease and related deaths are associated with witchcraft and not diabetes complications.

*When someone dies suddenly, they don’t know that the cause is diabetes but they think he/she has been bewitched …* (P5, male patient Malawi)

In conjunction with these traditional beliefs, the educators and HCPs reported that traditional medicine constitutes oftentimes a barrier to appropriate self-management of T2DM.

*One of our struggles here is that we have these cultural beliefs, and most of the patients also go to traditional peers, and they take natural medicine, so that can be an issue dealing with patients* (P1, female HCP, Mozambique)

This was further confirmed by the patient participants, many of whom experimented with traditional treatment before seeking medical care.

*There are bottles on the market which contain onions, garlic, and ginger and the suppliers say that if you take this, diabetes will come to an end* (P8, female patient, Malawi)

The participants felt encouraged that EXTEND could help overcome this barrier by educating the importance of clinical medicine.

*This education programme (EXTEND) is very important. It is there to encourage T2DM patients to stick to their medicine and see the importance of their treatment* (P2, male HCP, Malawi)

Religion and faith appeared to play a role in the management of the disease in Malawi. According to the patient participants, people may be misinformed in places of worship. Religion is perceived more trustworthy than medical advice, which can have detrimental consequences.

*Someone went for prayers and was told to stop medication but she later died* (P1, female patient, Malawi)

Societal beliefs about treatments, namely insulin, were also perceived as barriers to optimal diabetes management and important to consider when implementing. Patients and educators in Maputo, highlighted the taboo of insulin injections, which can hinder patients’ treatment. The EXTEND programme addressed these misconceptions.

*There’s this taboo in the community, and the patient won’t take insulin because of the reaction of their community. So, this is something that later on with the (EXTEND) classes, they started to understand why they must take insulin …* (P3, female educator, Mozambique)

#### Interpersonal influences

##### Benefits to and influences of family

Focus groups in both sites highlighted the benefit of the EXTEND programme not just for the patients themselves but also for their families and communities. Indeed, after participating in the EXTEND programme, participants shared their new acquired knowledge with their relatives and friends, with positive effects according to them.

*After the training (EXTEND) I joined my family, gave them a talk about the way of preparing meals and things have already changed in my house* (P5, male patient, Mozambique)

*I gave this to my husband who read the information by himself and whenever I forget, he reminds me what to do, he gives me full support* (P3, female patient Malawi)

In addition to sharing knowledge with partners, participants raised the importance to inviting family and guardians in future education programmes, as they played a significant role with the management of diabetes.

*Since the preparation of the food is often the wife or the aunt...who does it, they suggested that the next few times perhaps bringing someone they think could help in monitoring the food* (P3, female educator Mozambique)

#### ORGANISATIONAL influences

##### Diabetes training

While courses are available for communicable diseases, such training opportunities for NCDs and T2DM in both sites were extremely limited at an undergraduate and postgraduate level. HCPs in Maputo strongly believed that similar courses should be accessible.

*We used to have a course for HIV, but given that diabetes is an issue now, I would recommend strongly that our students are trained about how to deal with diabetes. At least of the level of secondary, tertiary and even quaternary level of hospitals.* (P4, male HCP, Mozambique)

Special postgraduate training for NCD was considered important for HCPs to build on knowledge and consultation skills to treat T2DM. Whilst diabetes training was available at certain localities outside Maputo and Malawi, educational opportunities for clinicians in diabetes management were extremely limited.

*Definitely none of us were trained with that kind of course* (P4, male HCP, Mozambique).

The lack of training in diabetes care also included the important aspects of patient self-management that was neglected by some HCPs.

*And the surgeons, they don’t understand … For them it (diabetes self-management) is a waste of time* (P1, female stakeholder, Mozambique)

However, one stakeholder in Maputo mentioned existing events that take place in order to improve diabetes knowledge among HCPs.

*What we decided to do in order to increase access to diabetes is to design a mandatory training program that anyone at each level can at least do the basic care* (P2, male stakeholder, Mozambique)

As for the patients, they felt diabetes knowledge was inconsistent across care, with oftentimes contradicting messages, which created confusion. To ensure that messages shared with patients remained consistent, suggestions were made to deliver diabetes training to doctors and other HCPs including dietitians.

*We get to the hospital and the doctor tells me, "eat every two hours," I go to another hospital, they say "eat every four hours." Sometimes they make us confused* (P4, female patient, Mozambique)

It was clear from patients’ testimonies that specialist training was required to improve HCPs’ consultation and emotional management skills.

*We were advised that a diabetic patient must not feel depressed, so that day when I visited the clinic I got tested and my glucose level was high, so the clinician shouted at me, he said “ your glucose level is too high, and it’s your fault take this medicine and go”* (P10, male patient, Malawi)

Participants viewed EXTEND as a programme for patients, but also for HCPs, to educate clinicians and doctors treating diabetes.

*The programme (EXTEND) could be more developed, could even be incorporated into college topics, nursing education* (P3, female educator, Mozambique)

##### Role of deciders and the government

For participants in both sites, the burden of diabetes needed to become a priority to the ‘deciders’, who were described as *‘those with political power, the Ministry of Health, the faculty, the university and scientific institutions*’.

*We need to ensure that it [diabetes] is a priority. For us it is a priority* (P3, female HCP, Mozambique)

*The government should really help us, we have seen cancer patients having a facility so the same should apply to us or is it that the government lack funding from donors?* (P11, female community participant, Malawi)

These deciders were portrayed by participants as crucial to the implementation of DSMES. A need for advocacy amongst the deciders to make diabetes a priority was reported. One stakeholder from Maputo believed there should be less focus on evidence, because these are already widely known to the deciders, instead there should be a “*collective move to change the current situation*” of diabetes.

*I don’t see a need for advocacy for funders … Everyone knows that, but there’s no move. There’s not a great move to make sure that we address properly the problem of diabetes … The economic impact of diabetes is like 10 times of the malaria* (P2, male stakeholder, Mozambique)

#### Community influences

##### Reach out

Similar to other chronic conditions, such as hypertension, participants suggested the use of media to reach out to communities and air education programmes on radios and televisions.

*We have different health programmes already like BP (blood pressure), HIV (human immunodeficiency virus), so diabetes should be on radio and TV stations too* (P5, male patient Malawi)

For stakeholders in Malawi the idea of using the radio was appealing, however, the issue of lack of funding and resources, appeared to be a significant barrier to implement education through radio and TV.

*The only problem is lack of resources but we would have loved to engage community radios to have these education programs aired on the radios* (P3, male stakeholder, Malawi)

With mobile phones becoming more accessible in SSA cities, various social media apps like Whatsapp were recommended as suitable modes of communication.

*If this can come through WhatsApp they will take them and study them by doing research to see if such things can be delivered to people and help them* (P8, female patient, Malawi)

Other suggestions to reach out to communities and implement programmes like EXTEND was to “*work with village chiefs*”, who can provide information about diabetes at schools and community gatherings and advise people to visit the hospital if needed.

*If we can make plans to reach other people, for example if you go through the village chiefs and tell them to gather the people for us; educators. Tell them that it’s about diabetes, then the chief will announce and gather the people to come at one place* (P2, male educator, Malawi)

#### Policy influences

##### Local guidelines on diabetes

Due to the fact that health centres are not responsible to treat diabetes, patients are referred to the central hospital.

*No as a facility (health centre) we don’t have any guideline for T2DM nor self-management education for T2DM.* (P3, male HCP, Malawi)

In Maputo, HCPs were not familiar with any local guidelines and emphasised the need for guidelines tailored to their local health system.

*We should talk and decide, are we using European (guidelines), American (guidelines), South African (guidelines), or similar to our environment or what are we going to do, so that we start talking the same language* (P5, male HCP, Mozambique)

The lack of ‘sharedlanguage’ in diabetes treatment and general clinical care was a main concern amongst HCPs in Maputo. Without a dialogue or communication amongst HCPs, patients would continue feeling confused.

*So, I think that we have to identify the main message to convey to patients, and once we have done that, all of us, we have to make sure that we are sending the very same message* (P4, male HCP, Mozambique)

##### NCD funding and resources

Relative to infectious diseases, T2DM lacks funding and resources. This has important repercussions including a limited availability and patient accessibility to vital medicines.

*In the end we are referred to the pharmacy to buy medicine on our own, which is expensive* (P7, *male* community participant, Malawi)

Participants expressed ‘*being ignored’* and requesting full focus on T2DM management. By collaborating with the Ministries of Health, participants felt that EXTEND could become part of wider diabetes care programmes and be implemented not only in hospitals but also in local health centres. HCPs advocated for collaboration from Ministries of Health to implement EXTEND into SSA wider diabetes care programme in both hospitals and local health centres alike.

*“There is need to use the Ministry of Health through NCD department. EXTEND should be established in all hospitals and health centres. It (EXTEND) should be training educators; nurses and volunteers to deliver EXTEND in all parts of the country* (P1, male HCP, Malawi)

The issue of funding was raised in both locations with regard to enhancing diabetes care in local and remote areas, and its implementation nationally. According to stakeholders in Malawi, several government bodies (such as “Diabetes Association of Malawi”, International Diabetes Federation” and “Development Communications Trust”), are already collaborating to promote diabetes care and diabetes education, and believe that there is a place for DSMES programmes like EXTEND.

*As a Ministry we are also partnering with our colleagues from World Diabetes Foundation. So what we are doing is teaching health workers how to manage this condition, community awareness of diabetes and providing patient education. So on patient education this is what we are discussing here, EXTEND is patient education and should be part of it* (P1, male stakeholder, Malawi)

##### Integrated and dedicated diabetes service in the SSA healthcare system

Delivering DSMES in clinics was unanimously agreed by participants. Whilst stakeholders shared similar views with educators that diabetes education must be integrated in clinics and communities, building a specialised diabetes clinic may be difficult due to the current structure and “*lack of infrastructures*” in the local health systems.

*Having a special room within the hospital for T2DM patients is a little bit problematic because hospital infrastructure is an issue* (P2, male stakeholder, Malawi)

The development of an integrated diabetes service, which would include DSMES, was suggested by both Lilongwe and Maputo groups. Despite their individual differences, both cities offer services for conditions such as tuberculosis, however, both locations lack the infrastructure to provide a dedicated service for diabetes where patients can be referred to and be treated by a specialist diabetes clinic.

*HIV has Lighthouse (specialist HIV support centre) and cancer has a hospital that is being constructed. We want the same to happen to T2DM. This place should have well trained doctors to attend to T2DM patients* (P7, male community participant, Malawi)

Similarly, in Maputo, participants suggested that a dedicated integrated diabetes service at their central hospital would benefit communities. Their vision is to integrate all areas of self-management and provide clinical and emotional support with the involvement of nutritionists, psychologists, diabetes specialist nurses and HCPs.

*We should first try to have a dedicated service just for diabetic patients, so that we can integrate all those areas like nutrition, maybe have psychologists … .And then, we should have an integrated service that any patient would come, even if they don’t have an appointment that day to see the doctor* (P4, male HCP, Mozambique)

## Discussion and conclusion

### Discussion

**Fig. 1:**
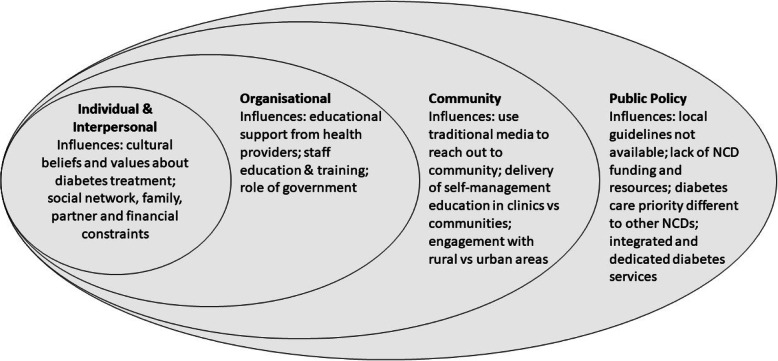
Findings from EXTEND qualitative study mapped onto the socio-ecological model: Influences to the implementation of DSMES programmes

This qualitative study explored factors that influence the implementation of DSMES programmes in LI-SSA countries, Malawi and Mozambique. We found that implementation of DSMES programmes are influenced by factors at multiple levels based on the socio-ecological framework (Fig. 1). The findings are critical for understanding if and how DSMES could be implemented and embedded in the T2DM services in urban and potentially rural areas in SSA countries.

### Individual and interpersonal influence

Our findings suggest that cultural beliefs, traditional medicine and healers play a pivotal role in diabetes self-management in Mozambique and Malawi health care. However, these can sometimes conflict with modern medicine and act as a barrier to disease self-management as highlighted in recent reports [19, 20]. In line with the Global Action Plan, we must recognise the cultural heritage of communities and respect traditional medicine accordingly to provide safer diabetes education [21]. Effective integration of traditional medicine would encourage an effective national response to diabetes self-management, involving key stakeholders such as religious institutions, traditional medicine practiotioners, communities and policy makers.

Societal beliefs and the stigma attached to diabetes management, including negative attitudes towards insulin, hinders an opportunity to self-care effectively. The taboo associated with insulin has been witnessed in other cultures, where taking insulin is associated with a number of myths and misconceptions [22], such being perceived as a drug user [23]. With the recent DSMES position statement in mind [24], DSMES must be designed to address not only people’s cultural needs, but also people’s health beliefs and emotional concerns living with T2DM. Thus, culturally adapted DSMES for SSA countries, would require addressing and minimising the hidden burdens experienced in LICs [25].

Our findings echo conclusions from previous studies indicating that people have poor level of knowledge regarding the importance of lifestyle behaviour and diabetes complications partially explaining why self-management in SSA remains poor [19]. Despite the willingness to manage T2DM**,** the ability to maintain optimal self-management was often influenced by the affordability of medication. This issue has been reported elsewhere suggesting a number of impediments faced when pursuing care, including personal and organisation cost and access of insulin [25–27]. Without the availability and affordability of key necessities for effective self-care, both DSMES and people with the condition are set up for failure. During the design and implementation of DSMES, it is important to consider these barriers to self-management, and appropriately tailor the education content and scope to people’s personal necessities and available resources.

### Community influence

Although our work was conducted in urban areas, it is recognised that DSMES delivery should be made available in rural areas that lack sophisticated facilities to support diabetes treatment [28, 29]. DSMES, such as EXTEND, could become available in rural and urban areas to ensure that patients regardless of location are not excluded from diabetes education, but are rather empowered to better manage their own condition.

This study suggests the use of traditional media, e.g. community radio and television, to reach out to all communities. In relation to DSMES, these methods would be an effective source of raising awareness and disseminating health information in local communities, as has been previously reported [30]. Thus, using the appropriate communication mode could help target a wider range of people cost-effectively, including young adults, older generations and the less educated [31].

Participants in our study discussed the importance to involve community leaders, i.e. village chiefs. If DSMES reach rural areas, we must be able to understand the values and beliefs of traditional communities. The involvement of community leaders aligns with a key Global Health Plan strategy for the prevention and control of NCDs, which states that organisations must engage with local communities to promote health and reduce NCD risk factors, through building community capacity and empowering key influential community figures [21]. This will not only help with building links between various sectors (community, private, academia), it will also build a strong infrastructure for the delivery of culturally adapted and community-led DSMES programmes.

### Organisational and policy influence to DSMES implementation

People with diabetes require appropriate and accurate resources and standardised guidance to adopt self-management behaviours. Currently however, there is a large unmet need for dedicated DSMES guidelines in SSA countries [11, 12, 30, 32]. Our findings highlight that these much needed national policies and local and national guidelines for diabetes management remain limited in SSA [33]. Without the availability of such standardised documents, DSMES are limited in content and consistency and are difficult to implement in real-world settings [11].

Limited HCPs diabetes training was also identified as a barrier in delivering effective diabetes care. The lack of diabetes knowledge from HCPs was attributed to a limited availability of training oportunities. Diabetes education is not easily available in SSA, as healthcare services are often stretched, restricting the provision of much needed high-quality education programmes [7, 25]. Key recommendation to address this issue,illustrated from our findings, is to provide DSMES in the form of diabetes education not only to people with diabetes, but to family members and clinicians also.

The participants showed major concerns in the lack of input from deciders in the implementation of diabetes education in LICs. The government and donors play a significant role in diabetes care and implementation of DSMES [21]. In collaboration, these bodies could support the development of infrastructures that include an integrated and dedicated diabetes service in the Mozambique and Malawi healthcare systems. This would involve a multi-disciplinary team including nutritionists, psychologists and diabetes specialist nurses. DSMES such as EXTEND would become a key component of diabetes services and as highlighted by our findings, such programmes would be provided in a hybrid form, adapted and implemented for people with T2DM and HCPs training.

#### Strengths and limitations

The analysis of the data was based on a systematic approach and the involvement of a second coder, allowed for reflective thoughts to ensure a level of dependability and confirmability. We provided a rich account of data including the interview topic guide, sample size and inclusion criteria to enhance transferability. As we restricted the study to urban areas, our findings may not be generalisable to rural LI-SSA. Future research could explore views from both urban and rural areas to understand the best approach for designing and implementing DSMES. The focus groups in Mozambique were conducted with an interpreter, which could have introduced bias. However, the research team consisted of Portuguese speaking researchers which would have minimised any issues around language. Although snowball sampling assisted the research team to recruit for all identified groups, we acknowledge that this recruitment technique may have certain shortcomings. For example, representativeness of the stakeholders and HCPs was relatively small.. Further research exploring stakeholders’ and HCPs’ needs and challenges would help address additional barriers to the implementation of DSMES. Both countries were selected due to pre-existing collaboration between partners, however, our findings illustrate different and common characteristics around the implementation of the EXTEND DSMES programme.

### Practice implications

This qualitative study will inform researchers and stakeholders of the key implications when considering the design and implementation of DSMES. Firstly, it is important to consider the impact of societal, cultural and religious beliefs when it comes to diabetes care in LICs in SSA. Misconceptions derived from traditional beliefs can have a detrimental effect on diabetes self-care. It is crucial to explore myths and misconceptions and gain a better understanding of what diabetes means to people living in LICs. Secondly, there is a need to develop an integrated and dedicated diabetes services in SSA healthcare systems, incorporating culturally adapted DSMES and tailored diabetes training for all professions involved in diabetes management (i.e. nutritionist, psychologist, nurses). We must ensure that a ‘shared language’ (referring to the same definitions, principles and standards of care) is adopted across SSA and endorsed by national guidelines specifically for DSMES. Thirdly, we must consider the role of the media and community leaders to help engage with communities and to support the wider infrastructure and implementation of DSMES programmes.

## Conclusions

Findings from this study suggest that multi-faceted factors (individual, community, organisational, policy) play a significant role to the implementation of DSMES programmes in Mozambique and Malawi. In the future, EXTEND could be incorporated in the development of diabetes training and dedicated diabetes services to act as an educational tool for both people with T2DM and HCPs in Mozambique and Malawi.

## Supplementary Information

**Additional file 1.**

## Data Availability

The data that support the findings of this study are available from the Leicester Diabetes Centre but restrictions apply to the availability of these data, which were used under license for the current study, and so are not publicly available. Data are however available from the authors upon reasonable request and with permission of the Leicester Diabetes Centre.

## Abbreviations

T2DM: Type 2 diabetes mellitus
LIC: Low-income countries
SSA: Sub-Saharan Africa
DSME: Diabetes self-management education
EXTEND: EXTending availability of self-management structured EducatioN programmes for people with type 2 Diabetes in low-to-middle income countries
HCP: Healthcare Professionals
NCD: Non-communicable diseases
HIC: High-income countries
