# Supplementary material for: Use of the socio-ecological model to explore factors that influence the implementation of a diabetes structured education programme (EXTEND project) inLilongwe, Malawi and Maputo, Mozambique: a qualitative study
Source: BMC Public Health. 2021 Jul 8;21:1355. doi: 10.1186/s12889-021-11338-y (PMC8268266; doi:10.1186/s12889-021-11338-y)
Supplement: Supplementary file 1 — Additional file 1. [file 12889_2021_11338_MOESM1_ESM.docx]

**Example of Topic Guide for EXTEND participants**

| **Possible areas of discussion**  **Venue / Group dynamic /Attendance**  Was the venue convenient for you?  Was it easy to attend?  Was there any reason you were unable to attend.  How comfortable did you feel in the group?  **The Education Programme**  What did you think of the education?  How did you find the format / delivery? (i.e. was straightforward, or easy to understand)  Did the programme meet your expectations? (If yes/no, prompt: In what way, can you give us an example)  How did you feel about the length/timing of the course?  How did you find the balance of information about different things like diet, physical activity and smoking?  Would you have liked to have family, friends, carers join you in the programme? Elaborate.  Can you tell us if you felt the sessions helped in any way to change your lifestyle? Prompt (i.e. in what way do you think, in terms of diet and physical activity)  Do you think other people would/could benefit from this education?  Do you think it should be available in your community? How do you think this could be available?  How would you improve the programme? |
| --- |
